# Transcription factors regulated by cAMP in smooth muscle of the myometrium at human parturition

**DOI:** 10.1042/BST20201173

**Published:** 2021-04-16

**Authors:** Jonathan K.H. Li, Pei F. Lai, Rachel M. Tribe, Mark R. Johnson

**Affiliations:** 1 Division of Reproductive and Developmental Biology, Department of Metabolism, Digestion and Reproduction, Imperial College London, London SW10 9NH, U.K.; 2 Department of Women and Children's Health, Kings College London, London SE1 7EH, U.K.

**Keywords:** bZIP transcription factors, cAMP, muscle contraction, pregnancy, signal transducers and activators of transcription, uterus

## Abstract

Cyclic adenosine monophosphate (cAMP) contributes to maintenance of a quiescent (relaxed) state in the myometrium (i.e. uterine smooth muscle) during pregnancy, which most commonly has been attributed to activation of protein kinase A (PKA). PKA-mediated phosphorylation of cytosolic contractile apparatus components in myometrial smooth muscle cells (mSMCs) are known to promote relaxation. Additionally, PKA also regulates nuclear transcription factor (TF) activity to control expression of genes important to the labour process; these are mostly involved in actin-myosin interactions, cell-to-cell connectivity and inflammation, all of which influence mSMC transition from a quiescent to a contractile (pro-labour) phenotype. This review focuses on the evidence that cAMP modulates the activity of TFs linked to pro-labour gene expression, predominantly cAMP response element (CRE) binding TFs, nuclear factor κB (NF-κB), activator protein 1 (AP-1) family and progesterone receptors (PRs). This review also considers the more recently described exchange protein directly activated by cAMP (EPAC) that may oppose the pro-quiescent effects of PKA, as well as explores findings from other cell types that have the potential to be of novel relevance to cAMP action on TF function in the myometrium.

## Introduction

In the uterus, smooth muscle cells of the myometrium (mSMCs) are maintained in a quiescent state throughout pregnancy to facilitate fetal growth and maturation. Labour typically starts at term (defined as ≥37 weeks of gestation), when the myometrium undergoes transition from quiescent to contractile state. This involves up-regulated expression/activity of contraction-associated proteins (CAPs), which include connexin-43, oxytocin receptor (*OXTR*) and sarcoplasmic/endoplasmic reticulum Ca^2+^ ATPase 2; changes in concentrations of prostaglandins and cytokines/chemokines also occur to indicate that labour is a pro-inflammatory process [1–3]. Sterile inflammation is suggested to increase within the myometrium, as pregnancy advances towards labour, to promote uterine contractions by up-regulating the expression of CAPs [4–6]. Spontaneous preterm (<37 weeks of gestation) labour (sPTL), a leading cause of mortality and morbidity of children under 5 years old [7,8], is linked to premature up-regulation of labour-related protein expression/activity; stimuli proposed to promote this include extrinsic inflammation (from infection), thrombin and mechanical stretch [9]. Our knowledge of the cellular processes involved in the spontaneous onset of labour are limited, especially with regards to the intracellular signalling events responsible for mSMC transition from a quiescent to contractile phenotype, which has hindered the development of effective therapies for preventing sPTL.

Transcription factor (TF) activity is expected to form an important connection between changes to *in utero* signals and mSMC phenotype adaptation. Among the most well-studied TFs for pregnancy/labour are nuclear factor κB (NF-κB), activator protein 1 (AP-1) and progesterone receptors (PRs; isoforms A and B). Regulation of these TFs in mSMCs is typically associated with pro-inflammatory mediator activation of mitogen-activated protein kinase (MAPK) signalling cascades. However, TF activity is also influenced by cyclic adenosine monophosphate (cAMP), which is a ubiquitous second messenger better known for promoting myometrial relaxation via its effects on contractile apparatus proteins by acting primarily through protein kinase A (PKA) [10–12]. The actions of cAMP can also be mediated via exchange protein directly activated by cAMP (EPAC; isoforms 1 and 2), which interacts with Ras-like small GTPase (Rap) proteins [13,14] to activate MAPKs [15] but its role in mSMCs is less understood. In this review, we will summarise established observations of labour-associated changes in abundance of cAMP signalling components in the myometrium, as well as discuss how they may impact on TFs known to bind to cAMP response element (CRE) sequences at gene promoters, along with the aforementioned labour-related TFs, to modulate contractility and inflammation.

## Dynamics of myometrial cAMP signalling

**Figure 1.**
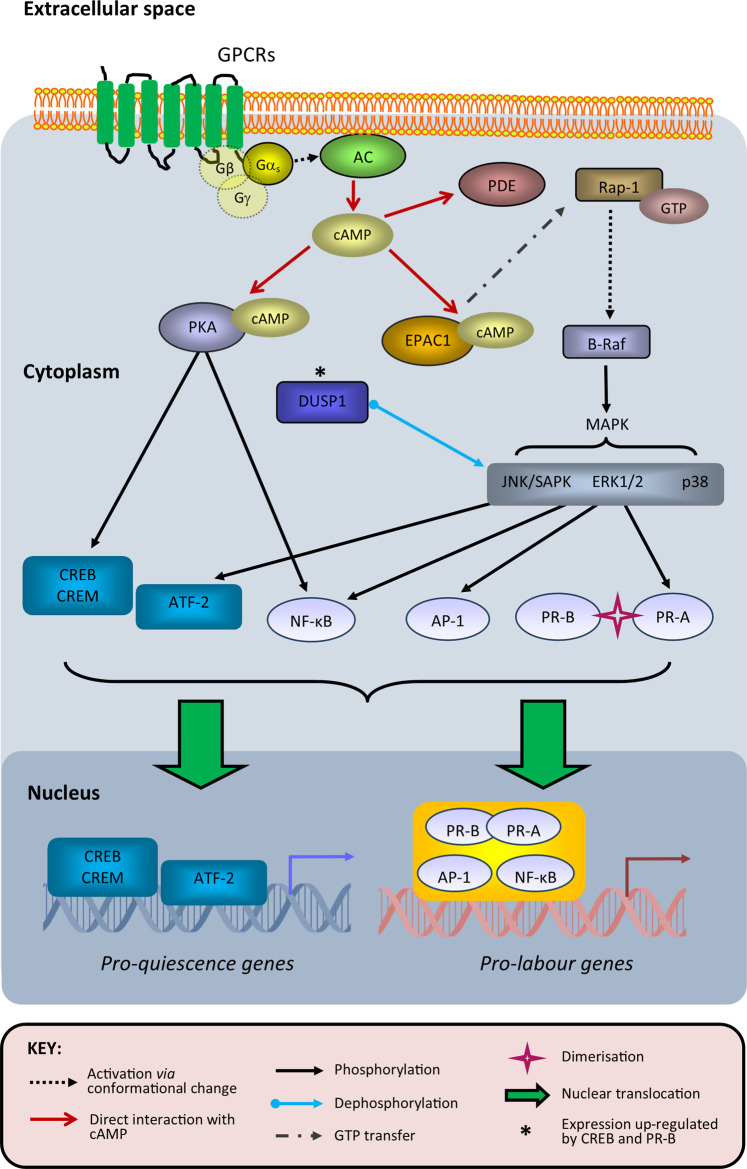
Overview of cAMP-regulated transcription factor activity in myometrial smooth muscle cells during pregnancy and labour. Upon agonist binding to G-protein coupled receptors (GPCRs) that promote cyclic adenosine monophosphate (cAMP) signalling, Gαs dissociates from its trimeric G-protein complex to activate adenylate cyclase (AC) and thus increase cAMP synthesis. Elevation of cAMP concentrations increase the probability of cAMP to bind both regulatory subunits of each tetrameric protein kinase A (PKA) complex, which causes the dissociation and activation of both its catalytic subunits that subsequently phosphorylate proteins with exposed serine/threonine-containing motifs compatible to their active sites. These include transcription factors that bind to the cAMP response element (CRE) sequence within compatible gene promoters, such as CRE-binding protein (CREB) and CRE modulator (CREM); cAMP-dependent transcription factor 2 (ATF-2) can bind to CRE sites but is not a known PKA substrate. CREB and CREM activities have been proposed to promote expression of pro-quiescence genes, and this is potentially influenced by CREB heterodimerisation with ATF-2. PKA (via CREB) and PR-B activity can enhance expression of dual specificity phosphatase 1 (DUSP1), which dephosphorylates mitogen-activated protein kinases (MAPKs) to reduce phosphorylation of their downstream targets; these include progesterone receptor A (PR-A), which is phosphorylated at its Ser344/345 residue by c-Jun N-terminal kinase (JNK)/stress-activated protein kinase (SAPK) to promote its heterodimerisation and subsequent transrepression of PR-B. In addition to PKA, exchange protein directly activated by cAMP 1 (EPAC1) can also bind cAMP, which leads to activation of guanosine triphosphate (GTP)-bound Ras-like small GTPase 1 (Rap1); this promotes the phosphorylation activity of B-Raf to increase downstream MAPK signalling via extracellular signal-regulated kinase 1/2 (ERK1/2), JNK, SAPK and/or p38. Subsequently, activator protein 1 (AP-1) and nuclear factor κB (NF-κB) transcription factors enhance the expression of pro-labour genes, which can involve interaction with PR heterodimers. PKA and EPAC1 activities are reduced by phosphodiesterase (PDE)-mediated degradation of cAMP.

The cAMP pathway is fundamental to all mammalian cells and primarily driven by G-protein coupled receptor (GPCR) activation, specifically through those coupled to either Gαs (cAMP-activating) or Gαi (cAMP-inhibiting) transducer proteins [16,17]. For mSMCs, the most recognised GPCRs involved in cAMP signalling during pregnancy/labour are β-adrenoreceptors [18] and prostaglandin E2 (PGE_2_) receptors [19]. The generic signal transduction steps that link GPCR–ligand interaction to cellular response is well described for many cell types [20,21] and their components that are relevant to our discussion of mSMCs are depicted in Figure 1. The potential importance of adenylate cyclase (AC; activated by Gαs to synthesise cAMP) and phosphodiesterase (PDE; hydrolyses cAMP to eliminate its activity) isoforms in shaping PKA-driven responses, as well as their likelihood of acting as drug targets, in the context of labour have been reviewed previously [22,23].

Known significant pregnancy and labour-associated changes in myometrial mRNA/protein abundance for cAMP signalling components are summarised in Table 1. With regards to their functional output, basal PKA and GPCR/Gαs-dependent AC activities, when specifically detected in membrane fractions prepared from myometrial tissues, are increased by pregnancy and subsequently decreased at labour [24–26], and activity ratios of cAMP-specific PDE4 isoforms are altered by pregnancy to sustain high cAMP concentrations in myometrial tissues [27,28]. Data from *in vitro* experiments with mSMCs have suggested that up-regulation of PDE4B may partly be mediated by increased pro-inflammatory interleukin (IL)-1β stimulation [29]. Together, existing expression and activity data supports the concept that pregnancy favours a high cAMP content within the myometrium to maintain quiescence by promoting PKA activity, which declines at labour. This is potentially paralleled by an increase in EPAC1 levels, which has been observed in mSMCs *in vitro* to be capable of enhancing oxytocin receptor (*OXTR*) expression [30] and thus promote a pro-contractile phenotype. Labour-related changes in expression/activity of TFs that can be influenced by PKA/EPAC have also been observed in human myometrium-derived samples and these will be discussed in the following sections.

**Table 1.** Changes in myometrial tissue expression of cAMP signalling components associated with human pregnancy and term gestation labour

Component of cAMP signalling	Pregnancy***^a^***		Labour***^b^***	
	mRNA	protein	mRNA	protein
Gαs	[-] [26]	[-] [25]; **⇑** [25,143]	[-] [30]	[-] [30]; **⇓** [25]
Adenylate cyclases (ACs)	n/a	**⇓ AC1, AC3, AC8, AC9** [144]**; ⇑ AC2, AC4, AC5, AC7** [144]	n/a	n/a
A-kinase anchoring proteins (AKAPs)	n/a	[-] **AKAP95,** [-] **AKAP79** [24]	↓ AKAP79 [30]	[-] AKAP79 [30]; [-] **AKAP95,** [-] **AKAP79** [24]**; ↓ AKAP79** [87]
Protein kinase A (PKA) – regulatory (R) subunits	↑ RIIα [24]	[-] RIα, ↑ RIIα [24]; **↑ RIIα** [24]	↓ RIIα [24,30]	[-] RIα [24], ↓ RIIα [24,30]; **↓ RIIα/β** [24,87]
Protein kinase A (PKA) – catalytic (C) subunits	n/a	[-] Cα, [-] Cβ [24]; **↑ Cα/β** [24]	n/a	[-] Cα, [-] Cβ [24]; **↓ Cα/β** [24,87]
Exchange protein directly activated by cAMP 1 (EPAC1)	n/a	n/a	↑ [30]	↑ [30]
cAMP-phosphodiesterases (PDEs)	[-] PDE4A, PDE4C, PDE4D [145]; ↑ PDE4B2 [145]	⇑ PDE4B, [=] PDE4D [27]	[-] PDE4B [30]	↓ PDE4B [30]

Associated references in brackets, where qualitative (⇓/⇑/[=]) or quantitative (↑/↓/[-]) findings presented were based on comparisons between ^a^pregnant (term gestation) vs non-pregnant and ^b^term labouring vs term non-labouring women (decrease (⇓↓)/increase (⇑↑)/no change ([=][-]) of former compared with latter for each pair of comparisons) for myometrial tissues obtained from the lower uterine segment at Caesarean section; n/a indicates the comparison has not been assessed; changes at protein level that were specifically observed in membrane fractions are shown in bold font.

## CRE-binding TFs

TFs that bind to the CRE nucleotide sequence (5′-TGACGTCA-3′) include CRE-binding protein (CREB), CRE modulator (CREM) and cAMP-dependent transcription factor (ATF), which are all members of the basic leucine zipper (bZIP) superfamily [31]. CREB is activated by PKA phosphorylation at its Ser133 residue to promote its nuclear translocation and dimerisation [32]. Active CREB dimers can interact with coregulators such as CREB-binding protein (CBP) or p300, which augment the ability of CREB to assemble transcriptional complexes at its target gene promoters by modifying chromatin structure via histone acetylation [33]. CREB protein abundance in human myometrium has been observed to decrease during pregnancy with no further change at labour [34]. Additionally, CBP abundance in myometrial tissues is increased during pregnancy and decreased at labour, whereas p300 is unchanged during both events [35]. Other CREB-binding partners, such as nuclear receptor sub-family 6, group A, member 1 (NR6A1) [36] and CREB-regulated transcription co-activator 1 (CRTC1) [37], have yet to be studied using mSMCs.

CREM is phosphorylated by PKA, which occurs specifically at the Ser117 residue of its CREMτ isoform [38]. In human myometrium, alternative splicing of CREM produces transcription activator (CREMτ2α) or repressor (CREMα) isoforms, which are decreased and increased, respectively, in their protein abundance both by pregnancy and, more so for CREMα, at labour [34,39]. This myometrial CREM isoform switch has been attributed to the activity of serine/arginine-rich splicing factor 5 (SRSF5; also known as SRp40), which decreases in its protein abundance during pregnancy and more so at labour; repression of its activity increases the presence of CREMα [40]. Microarray-based transcriptome analysis of mSMC cultures have shown that CREMτ2α and CREMα overexpression results in differential expression of 220 and 118 genes, respectively, only 16 of which were common between these two CREM isoforms; the same study also showed 958 genes were differentially expressed after CREB overexpression [39].

ATF-2 is the only member of the ATF family that has been detected at protein level in human myometrial tissues, in both its full-length form and a short ‘ATF2-sm’ variant [34] both of which were decreased in their protein abundance by pregnancy and, more so for full-length ATF-2, at labour. The same study also showed ATF2-sm expression was higher in upper (than lower) segment myometrium in pregnant (non-labouring and labouring) women, but they were of near-equal abundance in these two uterine regions in non-pregnant women. ATF-2 interaction with co-activators can be enhanced by CBP [41]. Phosphorylation of ATF-2 is more associated with stress-activated protein kinases (SAPKs) and c-Jun N-terminal kinases (JNKs) than PKA [42]. ATF-2 can bind to CRE as a homodimer but can also form heterodimers with Jun proteins; the latter bind with less affinity to CRE and cannot bind the AP-1 response element [43]. ATF-2 can also form heterodimers with CREB, thus forming an intersection between MAPKs and PKA for their regulation of CRE-containing gene promoters. The CRE-binding activity of ATF-2 in mSMCs has been demonstrated *in vitro* but endogenous labour-related gene targets for ATF-2 in the myometrium have yet to be determined [44].

## Combined effects of cAMP and progesterone on gene expression

Progesterone is well recognised for its pro-quiescent effects; classic labour-related genes that encode cyclooxygenase-2 (COX-2; *PTGS2*), connexin-43 (*GJA1*) and *OXTR* have been shown to be transcriptionally repressed by progesterone acting via PR-B during pregnancy [45,46]. With the onset of human labour, it has been proposed that pro-quiescent effects of PR-B are reduced by increased expression/activity of the transrepressive PR-A isoform, which forms heterodimers with PR-B to sequester it from promoter sequences to remove its suppressive effect on the expression of labour-related genes [47,48]. Other mechanisms for nuclear PR action mostly involve interactions with transcriptional coregulator proteins [49]; those proposed to contribute to progesterone-regulated gene expression in the myometrium for labour include GATA zinc finger domain-containing 2B (GATAD2B) [50], non-POU-domain-containing octamer binding protein (p54nrb) [51] and polypyrimidine tract-binding protein-associated-splicing factor (PSF) [52,53]. Regulation of mSMC progesterone content by 20α-hydroxysteroid dehydrogenase (20α-HSD) activity has also been considered as an important aspect of myometrial progesterone responsiveness at labour [46,54].

PR-A is 164 amino acids shorter than PR-B at its N-terminus [55], which results in the lack of an activation function (AF) domain (specifically AF3) that is present in PR-B and the first 140 residues of its N-terminus acts as a repressor region instead [56]. The canonical progesterone response element (PRE) sequence is 5′-ACAnnnTGT-3′ but PR can interact with promoter sequences that do not contain PREs [57,58]. Transrepression by PR-A requires sumoylation at its N-terminus [59]. In human mSMCs, pro-inflammatory stimulants lipopolysaccharide (LPS) and IL-1β can increase PR-A protein stability [60], which has been attributed to SAPK/JNK-mediated phosphorylation at its Ser344/345 residue [61,62]. Consequently, mSMC inflammation as a result of increased cytokines expression, which has been observed in myometrium biopsies from labouring (compared to non-labouring) women [63], is expected to reduce the pro-quiescent effect of PR-B at labour.

In relation to cAMP signalling, forskolin (AC agonist) can enhance the ability of progesterone to suppress IL-1β-induced *PTGS2* expression in human mSMC cultures [64]. This apparent synergistic effect against inflammation-driven transcriptional activity is likely to be mediated via modulation of MAPKs, whereby cAMP and progesterone together (via CREB [65] and PR-B [66], respectively) increase dual specificity phosphatase 1 (DUSP1) expression that represses SAPK/JNK activity [67,68] to reduce PR-A Ser344/345 phosphorylation [62]. PR-A and PR-B abundance has been observed to exist at a 1 : 1 ratio in myometrium biopsies from non-labouring pregnant women at term gestation [69,70]; when this is modelled *in vitro* using the human myometrium hTERT-HM^A/B^ cell line [48], treatment with a combination of forskolin and progesterone can maintain a transcriptome profile that resembles the *in vivo* non-labouring state more closely than treatment with either agent alone [71].

The endometrium is another uterine tissue where the effects of simultaneously up-regulating cAMP and progesterone signalling have been studied, mostly in the context of their ability to promote decidualisation when modelled in cell cultures using 8-bromo-cAMP (cAMP analogue) and medroxyprogesterone acetate (progestin). Here, this treatment combination increases Forkhead box protein O1 (FOXO1) TF expression and activity, which is needed to up-regulate IL-8 [72]; RNA-seq data suggests signal transducer and activator of transcription (STAT) TF activities are also increased at the same time, which is eventually replaced by NF-κB as expected *in vivo* prior to the end of the menstrual cycle [73]. Thus, cAMP and progesterone signalling together have been observed to promote opposing effects on inflammation in endometrial cells and mSMCs, at least where there are also differences in *in vitro* protocols and stage of reproduction.

## NF-κB

The NF-κB family of TFs consists of RelA (p65), RelB, c-Rel, p100/p52 and p105/p50 proteins, which form different combinations of dimers that bind to consensus sequence 5′-GGGRNYYYCC-3′ to control the expression of inflammation-related genes [74–76]. Various pregnancy/labour-related stresses (e.g. uterine stretch and pro-inflammatory cytokines) activate NF-κB in mSMCs [77,78]. The p65 : p50 complex, which is the most commonly explored NF-κB dimer for mSMCs, is inactive in the cytoplasm while bound to inhibitor of NF-κB (IκB). Pro-inflammatory stimuli, such as LPS, IL-1β and tumour necrosis factor α (TNFα), activate IκB kinases (IKKs) to promote IκB phosphorylation, which leads to its ubiquitination and subsequent proteolysis. This removal of IκB binding from p65 and p50 exposes their nuclear localisation sequence, which results in their translocation into the nucleus to bind promoter sequences of genes that include *CXCL8*, *CCL2*, *PTGS2* and *OXTR*.

A study that examined NF-κB protein abundance in myometrium biopsies from non-pregnant and pregnant (non-labouring and labouring) women showed levels of c-Rel, p105, p50 and p100 were reduced by pregnancy, while p65 (in lower, but not upper, uterine segment myometrium) was only reduced at labour [79]. Additionally, NF-κB dimer composition was found to change during pregnancy, from being predominantly p50 homodimers in non-pregnant women to being mostly p65 : p50 heterodimers in pregnant women; data from electromobility shift assays suggested that p65 : p50 DNA binding increases at labour despite a decrease in total p65 protein levels. The relevance of PKA to NF-κB activity was demonstrated in the same study; p65 was bound to PKA (catalytic subunits) and phosphorylated at its Ser536 residue in non-pregnant and pregnant women, most likely to maintain p65 in an inactive state prior to labour.

In other cell types, PKA is capable of phosphorylating p65 at its Ser276 residue [80], which can either activate NF-κB via enhanced CBP/p300 recruitment [81] or suppress its nuclear translocation [82]. In myometrium, it has been suggested that complex formation with PKA and IκBα together prevents p65 Ser276 phosphorylation in both non-pregnant and pregnant (non-labouring) women [79]. An alternative mechanism for cAMP-enhanced NF-κB activity has been proposed for human foetal lung epithelial cells, where miRNAs (specifically miR-199a and miR-214) that suppress NF-κB and COX-2 expression can be decreased in abundance by treatment with cAMP analogue, dibutyryl cAMP [83]; this was attributed to CREB-mediated reduction of zinc finger E-box-binding homeobox 1 (ZEB1) TF expression. Interestingly, progesterone is known to have the opposite effect on ZEB1 in mSMCs, which suppresses COX-2 expression (using the same miRNAs) during pregnancy [45].

The consequences of interactions between PKA and NF-κB on labour-related gene expression in the myometrium have not been directly assessed. Prolonged *in vitro* treatment of human mSMCs with forskolin has, however, been observed to increase COX-2 expression and PGE_2_ secretion by promoting MAPK activity [84]; although the functional impact of PGE_2_ can be either pro-relaxatory or pro-contractile depending on the expression levels of its receptor isoforms present [19,85,86]. The combination of forskolin and progesterone during IL-1β stimulation can also reduce p65 binding to the *PTGS2* promoter [64]. Overall, a labour-associated decrease in catalytic and regulatory PKA subunits in myometrium at term gestation [24,87] is expected to enhance pro-inflammatory genes expression driven by NF-κB.

Both enhancement and suppression of NF-κB by cAMP have been demonstrated more directly in other cell types and mostly attributed to PKA. Treating a human monocyte cell line with dibutyryl cAMP can increase gene promoter activity mediated by NF-κB, which is an observation used to explain how cholera toxin causes inflammation [88]; whereas nuclear translocation of NF-κB in brain (hippocampus and cortex) tissues of mice after *in vivo* LPS injection can be decreased by PDE4 inhibitor, rolipram [89]. In airway SMCs, the anti-inflammatory effects of PKA have been associated with enhanced cell proliferation [90,91]. Whereas for mSMCs, it has not been determined whether their proliferative state, which increases during hyperplasia at early pregnancy before switching to a hypertrophic state at late pregnancy to expand uterine size [92], influences the ability of PKA to regulate inflammation in the same way.

From the perspective of NF-κB regulating cAMP action, the *GNAS* (Gαs-encoding gene) promoter sequence is rich in GC nucleotides, which makes it ideal for binding TFs like CREB and NF-κB [93]. Indeed, TNFα-stimulated p65 activity can reduce *GNAS* promoter activity in mSMCs [94] by reducing CBP recruitment [95]. NF-κB is also activated by IL-1β and other pregnancy-related stress mediators; their combined ability to activate NF-κB has been proposed to progressively increase during advancing gestation [60], which eventually reaches a level that reduces cAMP-regulated gene expression (by reducing PKA activity) to promote labour [62]. Currently, it is not certain what the *in utero* order of events are at labour between the activities of cAMP/PKA and NF-κB in mSMCs.

## AP-1

Active AP-1 dimers are formed from Jun (c-Jun, JunB and JunD) and Fos (c-Fos, FosB, Fra-1 and Fra-2) proteins, which can also dimerise with members of the ATF and Jun dimerisation protein (JDP) families, to function as bZIP TFs [96]; the consensus AP-1 binding sequence is 5′-TGA(C/G)TCA-3′ [97]. AP-1 activation requires its phosphorylation by MAPKs [98] that include members of the JNK family, which can be regulated by cAMP via EPAC (discussed below) as demonstrated in vascular SMCs [99]. Expression of c-Fos and c-Jun can be increased by CREB, which involves its phosphorylation by extracellular signal-regulated kinase (ERK)1/2 when demonstrated in a mouse-derived monocyte/macrophage cell line stimulated with IL-33 [100]. Comparisons for protein abundance of each Jun and Fos protein in the myometrium between non-labouring and labouring women have been undertaken for whole tissue extracts [101] and nuclear/cytosolic fractions [102], which demonstrated that they are differentially altered by labour (i.e. not all AP-1 monomers were increased or decreased to the same extents as each other). AP-1 can interact with PR to modulate *GJA1* transcription in mSMCs [54]. Here, the activity of AP-1 has been proposed to be repressed by PR-B and activated by PR-A, which suggests that a labour-related increase in PR-A expression [47,48] can enhance AP-1 activity to increase connexin-43 abundance. The ability of PKA to regulate PR-A activity (discussed above) presents a potential layer of complexity to their relationship with AP-1 in mSMCs. Promoter sequences of some labour-related genes contain binding sites for both AP-1 and NF-κB [103–105], and these TFs can also interact with each other to promote labour-related changes [106]; how this impacts on their interaction with cAMP signalling in mSMCs is not yet known.

## EPAC and its MAPK-related role

EPAC1 is expressed in a wide range of tissues and believed to be ubiquitous [13]. EPAC2 has so far been observed as more restricted in its expression profile, whereby it has only been detected in the brain, as well as neuroendocrine and endocrine systems [107]. Their roles are likely to differ depending on cell type and micro/nano-domain localisation [108]. For example, mouse genetic knockout models have demonstrated that EPAC1 is mostly anti-inflammatory, while EPAC2 is pro-inflammatory, specifically in lung tissues [109]. PKA and EPAC have similar affinities for cAMP according to enzymology experiments [14,110], although recent cell-based data obtained using fluorescent cAMP biosensors question the physiological relevance of these findings [111,112]. Nevertheless, the assembly of different cAMP effector combinations in discrete micro/nano-domain regions of the intracellular environment is expected to allow compartmentalised control of PKA and EPAC activities [113–115]. There is certainly a deficit in our knowledge for this aspect of cAMP signalling in mSMCs, which needs to be explored to at least the same extent as it has been in other organ systems [116–118] for us to fully assess their roles in regulating uterine contractility and inflammation during pregnancy/labour.

Cellular stresses during pregnancy in mSMCs can activate MAPK signalling and be inhibited by PKA activation. However, as mentioned above, *in vitro* forskolin stimulation to enhance cAMP accumulation for a prolonged period of time can activate MAPKs [84]. The latter is potentially attributable to EPAC, which activates Rap proteins upon cAMP binding to enhance activities of MAPKs (via phosphorylation by B-Raf) that include ERK1/2, MAPK/ERK kinase (MEK)1/2 and p38 [119,120]. EPAC1 abundance in myometrium biopsies from term pregnant women has been observed to increase during the early phase of labour [30]. Although *in vitro* RNA interference-based silencing of EPAC1 expression in human mSMCs has demonstrated that this cAMP effector does not contribute to COX-2 up-regulation promoted by forskolin [84]; similar experiments have instead demonstrated that EPAC1 can increase *OXTR* expression when PKA is suppressed [30]. So far, these are the only studies that have examined the role of EPAC1 in mSMCs and none are available for EPAC2. EPAC1/2 roles in endometrial stromal cells have also only recently been of interest, where they have been proposed to enhance PKA-driven prolactin (*PRL*) gene promoter activity during decidualisation [121], and mediate cAMP-dependent (but PKA-independent) ERK1/2 phosphorylation promoted by human chorionic gonadotropin [122].

Future use of highly specific EPAC activators, such as 8-pCPT-2′-O-Me-cAMP [119], and inhibitors will be useful in evaluating the importance of EPAC in human mSMCs at labour; this approach has provided valuable information from studies on EPAC action in cardiovascular and respiratory SMCs with regards to their response to stress stimuli [123,124]. For example, 8-pCPT-2′-O-Me-cAMP treatment of wild-type mice-derived aortic SMCs can decrease cofilin phosphorylation to reduce stress fibre formation, which decreases lamellipodia formation needed for vascular remodelling, and this does not occur in aortic SMCs from EPAC1 knockout mice [125]. Treatment of immortalised human bronchial SMCs with either 6-Bnz-cAMP, a PKA-specific activator, or 8-pCPT-2′-O-Me-cAMP can inhibit IL-8 secretion in response to cigarette smoke extract (CSE), but PKA and EPAC distinctly prevent CSE-mediated decrease in IκBα protein abundance and increase in ERK1/2 phosphorylation, respectively [126]. A novel EPAC1-specific inhibitor, AM-001, has recently been shown *in vivo* to prevent murine cardiac fibrosis, immune cell infiltration and myocyte enhancer factor 2 (MEF2) TF activation in response to prolonged β-adrenoreceptor activation [127].

## Cross-talk between cAMP and other messengers

Each TF and their complexes often act as hubs for multiple signalling pathways that are simultaneously activated by numerous combinations of cell surface receptors, which are predominantly GPCRs and receptor tyrosine kinases [128–130]. In addition to cAMP and MAPKs, Ca^2+^ signalling is important to mSMC function and can regulate TFs [131]; oxytocin and PGF_2α_ receptor activation increases myometrial Ca^2+^ mobilisation to promote contractility. Along with MAPKs, Ca^2+^ can also influence cAMP accumulation by regulating PDE activity [132]. In contrast, cyclic guanosine monophosphate (cGMP) has been proposed to have a lesser impact on mSMCs at labour due to lack of effect on their contractions [133]. Nitric oxide can promote myometrial relaxation but a nitrosoproteome analysis of uterine biopsies from pregnant women did not identify cAMP-related TFs of interest as labour-associated nitrosylation targets [134].

CREB Ser133 phosphorylation can be increased by Ca^2+^-calmodulin-dependent protein kinase IV (CaMKIV), along with MAPKs [135] and protein kinase G (PKG) [136], as demonstrated in other cell types; it can also be decreased by Ca^2+^-activated calcineurin. NF-κB nuclear translocation can be increased by inhibiting large-conductance Ca^2+^-activated K^+^ (BK) channels in mSMCs, which has been proposed to involve IKK activity [137], whereas CaMKIV-driven p65 phosphorylation can promote its transactivation and interaction with CBP in HeLa cells [138]. Members of the AP-1 family can form complexes with those of the nuclear factor of activated T-cells (NFAT) family of calcineurin-dependent TFs, which can also be phosphorylated by MAPKs [139] and multiple NFAT isoforms are expressed in human myometrium during pregnancy [140].

Together, observations like these exemplify the complexity that needs to be considered when working towards identifying upstream factors of TFs that are specifically responsible for promoting myometrial contractions to initiate labour. Various stimuli may promote labour [141], but their relative importance and order of interactions remain to be determined. Integrative analyses of the aforementioned and future data for myometrial cAMP actions with those for other intracellular messengers are needed to build a complete picture of mSMC signalling networks [142]. From this approach, the sequence of cellular events critical to the transition from end of pregnancy to the beginning of labour are more likely to be identified.

## Summary

To date, studies related to cAMP in the myometrium have assessed its regulation of cytosolic contractile apparatus function more than its effects on nuclear TF activity. Data for the latter mostly demonstrate how cAMP, either alone or in partnership with progesterone, can modulate labour-related gene expression to complement PKA pro-relaxation effects at the contractile apparatus and/or regulate inflammation. Instead of examining aspects of myometrial cAMP signalling for each TF separately, future work should focus on how the connections between them are altered to result in myometrial activation for initiating labour. Combining this with in-depth consideration of cAMP spatio-temporal organisation, the role of EPAC and cross-talk with other intracellular messengers, will help us to identify new ways to clinically modulate its activity to reduce the occurrence of pregnancy/labour-related complications.

## Perspectives

- Modulation of gene expression by TF activity is commonly investigated to gain insight into how the myometrium (uterine smooth muscle) transitions from a quiescent to contractile phenotype for the labour process to be initiated. TFs regulated by cAMP action have been shown to influence the expression of several classic labour-related proteins (mostly involved in contraction and inflammation) and thus likely to influence the success or failure of uterine contractions that lead to childbirth.
- Cytosolic actions of cAMP are best known for promoting myometrial relaxation by acting on the contractile apparatus via PKA, but cAMP can also control nuclear DNA-binding activities of myometrial TFs at labour-related gene promoters. The expression/activities of several proteins involved in cAMP synthesis/degradation and subsequent PKA activation are up-regulated by pregnancy and down-regulated at labour.
- Future efforts in identifying ways to harness the uterine pro-quiescent effects of cAMP will need to focus on the importance of spatio-temporal factors of cAMP signalling, as well as the activities of cAMP-binding proteins other than PKA, in mSMCs. A better understanding of how cAMP controls myometrial contractions will aid the development of therapies that reduce maternal and neonatal risk from obstetric complications, which includes preterm birth.

## Open Access

Open access for this article was enabled by the participation of Imperial College London in an all-inclusive *Read & Publish* pilot with Portland Press and the Biochemical Society under a transformative agreement with JISC.

## Abbreviations

20α-HSD: 20α-hydroxysteroid dehydrogenase
AC: adenylate cyclase
AF: activation function
AP-1: activator protein 1
ATF: cAMP-dependent transcription factor
BK: large-conductance Ca^2+^-activated K^+^
bZIP: basic leucine zipper
CaMK: calmodulin-dependent protein kinase
cAMP: cyclic adenosine monophosphate
CBP: CREB-binding protein
cGMP: cyclic guanosine monophosphate
COX-2: cyclooxygenase-2
CRE: cAMP response element
CREB: CRE-binding protein
CREM: CRE modulator
CRTC1: CREB-regulated transcription co-activator 1
CSE: cigarette smoke extract
DUSP1: dual specificity phosphatase 1
EPAC: exchange protein activated by cAMP
ERK: extracellular signal-regulated kinase
FOXO1: Forkhead box protein O1
GATAD2B: GATA zinc finger domain-containing 2B
GPCR: G-protein coupled receptor
GTP: guanosine triphosphate
IKK: IκB kinase
IL: interleukin
IκB: inhibitor of NF-κB
JDP: Jun dimerisation protein
JNK: c-Jun N-terminal kinase
LPS: lipopolysaccharide
MAPK: mitogen-activated protein kinase
MEF2: myocyte enhancer factor 2
MEK: MAPK/ERK kinase
mSMC: myometrial smooth muscle cell
NFAT: nuclear factor of activated T-cells
NF-κB: nuclear factor κB
NR6A1: nuclear receptor sub-family 6, group A, member 1
p54nrb: non-POU-domain-containing octamer binding protein
PDE: phosphodiesterase
PGE_2_: prostaglandin E2
PKA: protein kinase A
PKG: protein kinase G
PR: progesterone receptor
PSF: polypyrimidine tract-binding protein-associated-splicing factor
Rap: Ras-like small GTPase
SAPK: stress-activated protein kinase
sPTL: spontaneous preterm labour
SRSF5: serine/arginine-rich splicing factor 5
STAT: signal transducer and activator of transcription
TF: transcription factor
TNFɑ: tumour necrosis factor α
ZEB1: zinc finger E-box-binding homeobox1
